# RNF43 R117fs mutant positively regulates Wnt/β-catenin signaling by failing to internalize FZD expressed on the cell surface

**DOI:** 10.1038/s41598-022-10868-8

**Published:** 2022-04-29

**Authors:** A-Ri Cho, Hee Jung Sul, Yoo Jin Kim, Bohyun Kim, Dae Young Zang

**Affiliations:** 1 Hallym Translational Research Institute, Hallym University Sacred Heart Hospital, Anyang-si, 14054 Republic of Korea; 2 Division of Hematology-Oncology, Department of Internal Medicine, Hallym University Sacred Heart Hospital, Hallym University College of Medicine, 22, Gwanpyeong-ro 170, beon-gil, Dongan-gu, Anyang‑si, Gyeonggi‑do 14068 Republic of Korea

**Keywords:** Cancer, Cell biology, Oncology

## Abstract

RING finger protein 43 (RNF43) encodes the transmembrane E3 ubiquitin ligase, which targets the Wnt receptor Frizzled (FZD). RNF43 mutations have been discovered in various human cancers including colon, pancreatic, stomach, ovarian, and liver cancers. Functional studies on RNF43 missense mutations have shown that they negatively regulate Wnt signaling; however, there are few functional studies on RNF43 frameshift mutations. In this study, we showed that R117fs and P441fs mutants enhanced Wnt/β-catenin signaling, whereas Q409fs and G659fs mutants retained the ability to suppress Wnt/β-catenin signaling. Specifically, R117fs was unable to ubiquitinate FZD5 due to lack of the RING domain, although it was able to interact with FZD5. Immunofluorescence showed that R117fs failed to internalize FZD5 expressed on the cell surface. We also showed that LGK974, a potent Wnt inhibitor, decreased the Wnt/β-catenin activity by R117fs and P441fs mutations. Together, these results demonstrate that RNF43 frameshift mutations retain normal functionality; thus, targeted anti-cancer therapy can be developed according to the mutation type of RNF43.

## Introduction

Wnt signaling plays critical roles in the regulation of diverse processes including cell proliferation, survival, migration, and polarity. The Wnt ligand binds to its two receptors, Frizzled (FZD) and low-density lipoprotein-related receptors 5 and 6, to initiate intracellular signaling via β-catenin. In the absence of binding between Wnt ligand and its receptors, cytoplasmic β-catenin is targeted by a degradation complex composed of adenomatous polyposis coli (APC), Axin, and glycogen synthase kinase 3 α/β^1^. Mutations of Wnt pathway components, such as APC, β-catenin, and Axin 1, result in aberrant activation of Wnt/β-catenin signaling. These mutations have been identified in various tumors and diseases^2,3^; therefore, precise regulation of Wnt signaling is important.

Ubiquitin-mediated regulation plays an essential role in Wnt signaling^4^. RING finger protein 43 (RNF43) is an E3 ligase that acts as a negative regulator of Wnt signaling by inducing degradation of the Wnt receptor FZD^5,6^. RNF43 mutations have been identified in various cancers, such as colorectal adenocarcinoma, pancreatic cancer, and gastric cancer (GC)^7^. The vast majority of RNF43 mutations are deletions or insertions^8^. We identified five frameshift mutations in patients with GC. Previous studies have used missense mutations to elucidate the functional role of RNF43; however, there have been no reports on the function of frameshift mutations.

In this study, we cloned the mutant types identified in GC patients and investigated whether truncated proteins due to frameshift mutations affect Wnt signaling. The results showed that R117fs is a positive regulator; R337*, Q409fs, and G659fs are negative regulators; and P441fs is a partial positive regulator of Wnt signaling. The positive effects induced by R117fs and P441fs were inhibited by the Wnt inhibitor LGK974, indicating that R117fs and P441fs are potential targets for Wnt inhibitors.

## Results

### RNF43 R117fs and P441fs mutants lost the negative regulator function of RNF43 on Wnt/β-catenin signaling

**Figure 1:**
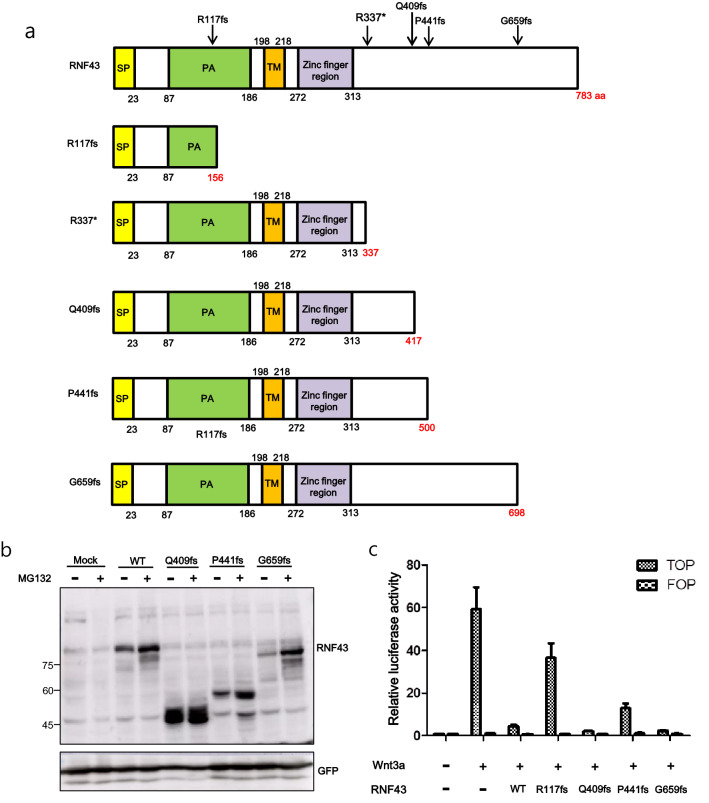
R117fs and P441fs mutants identified in GC patients lost the negative regulator function of RNF43 on Wnt/β-catenin signaling. (**a**) Distribution and types of RNF43 mutations. The conserved domain was from UniProt. SP, signal peptide; PA, protease-associated domain; TM, transmembrane. The a.a created by frameshift is represented in the domain. (**b**) Expression of WT RNF43 and mutant constructs. Ubiquitination of RNF43 constructs was evaluated upon MG132 treatment. (**c**) The effects of WT RNF43 and its mutants on Wnt/β-catenin signaling were examined using the TOP/FOP luciferase reporter assay. WT RNF43 suppressed the Wnt/β-catenin signaling induced by Wnt3A. R117fs and P441fs mutants lost the suppressor function of RNF43 in the Wnt/β-catenin signaling pathway. Q409fs and G659fs mutants exerted a negative regulatory function in WT RNF43.

We identified RNF43 truncating mutations, including R117fs, R337*, Q409fs, P441fs, and G659fs, in Korean patients with GC. These mutations have also been reported in various cancers, including in The Cancer Genome Atlas (Supplementary Fig. S1)^9^. To determine the function of these RNF43 truncating mutations, we cloned WT RNF43 and four truncating mutations (R117fs, Q409fs, P441fs, and G659fs) into expression plasmids, followed by transfection into HEK293T cells and subsequent western blot analysis with RNF43 antibody to detect the corresponding protein expression (Fig. 1a, b).

Next, we examined whether truncation by frameshift destabilized the RNF43 protein. To this end, we treated HEK293T cells overexpressing WT RNF43 or the truncated mutants with MG132, a proteasome inhibitor. As shown in Fig. 1b, we observed that the G659fs was expressed at lower level than WT RNF43.MG132 treatment increased the protein levels of WT RNF43 and the truncated mutants. Also, we generated C-terminal FLAG-tagged R117fs because anti-RNF43 antibody recognizes amino acids 252–276 of human RNF43. As shown in Fig. S2, MG132 did not increase the protein expression of R117fs. Thus, truncated proteins with the exception of R117fs and WT RNF43 were degraded via the proteasome pathway. Next, we examined the function of the RNF43 truncating mutations in the Wnt/β-catenin signaling pathway using HEK293T cells transfected with the TOP/FOP Flash luciferase reporter. Consistent with the function of RNF43, overexpression of WT RNF43 decreased exogenous Wnt3a-induced activation (Fig. 1c). We expected that the four truncating mutations would not inhibit Wnt signaling, because frameshift mutations lead to protein truncation, resulting in oncogenic loss-of-function mutations or dominantly acting oncogenes^10^. However, interestingly, R117fs and P441fs mutations showed a reduced inhibitory activity on the canonical Wnt pathway compared with WT RNF43 in the presence of exogenous Wnt3a, whereas other mutations suppressed Wnt/β-catenin signaling (Fig. 1c). We expected that the G659fs mutation would lead to loss of repressor function of Wnt signaling because of its high frequency of occurrence in tumors. Also, overexpressed P441fs had a partial dominant-negative effect.

### LGK974 suppresses Wnt/β-catenin signaling induced by R117fs and P441fs

**Figure 2:**
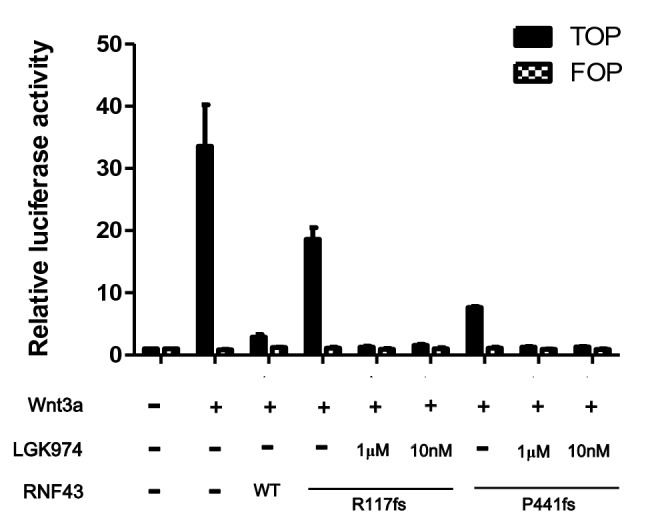
LGK974 inhibits the enhanced Wnt/β-catenin signaling pathway activity induced by R117fs and P441fs mutants. LGK974, a highly specific and potent Porcupine inhibitor, effectively inhibited Wnt/β-catenin signaling at a low concentration of 10 nM.

Next, we determined whether LGK974, a Porcupine inhibitor that specifically inhibits Wnt secretion^11^, could inhibit Wnt/β-catenin signaling induced by the RNF43 truncating mutations. HEK293T cells transfected with R117fs or P441fs were treated with LGK974 (10 nM or 1 μM). As shown in Fig. 2, LGK974 (10 nM) potently decreased the activity of Wnt/β-catenin induced by R117fs or P441fs. These effects were more potent than the repressive effect of WT RNF43.

### RNF43 R117fs do not decrease FZD5 expression

**Figure 3:**
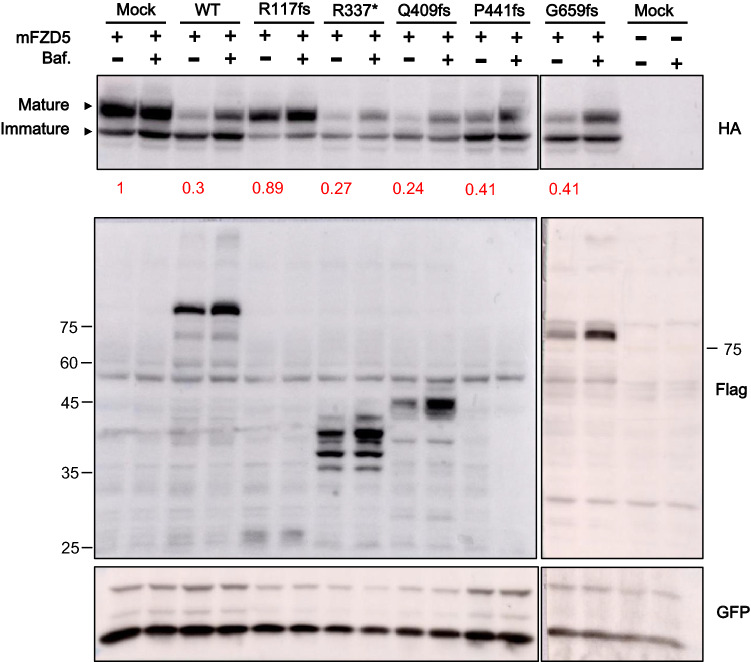
WT and mutant RNF43 regulate protein expression of the FZD receptor. WT RNF43 downregulated the protein expression of mFZD5. R117s overexpression had no effect on mFZD5 protein expression. Treatment of cells with bafilomycin A1 (Baf) rescued FZD5 protein expression from WT RNF43, and other RNF43 mutants mediated downregulation. The protein levels of mature FZD5 were quantified using Image J and the values are represented below the band.

Previous studies have shown that RNF43 acts as an inhibitor of Wnt signaling by reducing expression of the FZD receptor on the cell surface^5,12^. Therefore, we evaluated the effects of WT RNF43 and RNF43 truncated mutants on FZD5 expression by western blot analysis. We co-transfected C-terminal FLAG-tagged WT RNF43 or RNF43 truncated mutants with mFZD5-HA in HEK293T cells. Consistent with previous studies, overexpression of WT RNF43 selectively reduced the expression of mature mFZD5, the complex glycosylated form of FZD5, whereas the immature form remained unaffected^5^. However, treatment with bafilomycin A1, an inhibitor of autophagosome-lysosome fusion, prevented the degradation of mFZD5 by WT RNF43 (Fig. 3). The overexpression of R117fs had no effect on the mature and immature forms of mFZD5. Interestingly, overexpression of the truncated mutants R337*, Q409fs, P441fs, and G659fs selectively reduced the expression of mature FZD5, although P441fs slightly increased the activity of Wnt/β-catenin signaling compared with WT RNF43 (Figs. 1c, 2). These decreases were rescued by treatment with bafilomycin A1. Together, these observations suggest that the RNF43 truncating mutations R337*, Q409fs, and G659fs inhibited Wnt/β-catenin signaling by decreasing the expression of FZD, whereas R117fs caused loss of the suppressive activity of RNF43.

### RNF43 R117fs mutant does not decrease FZD5 on the cell surface

**Figure 4:**
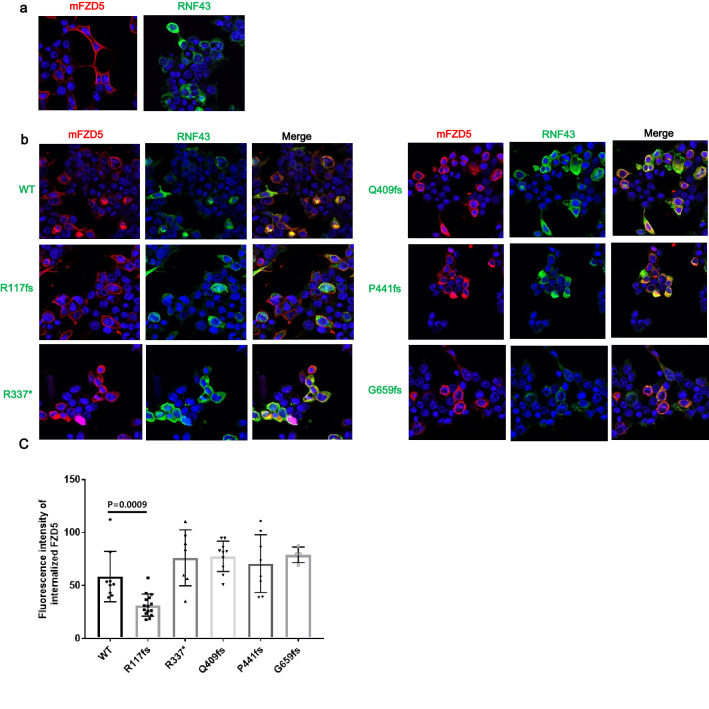
R117fs mutant does not downregulate cell surface expression of FZD. (**a**) Subcellular localization of Myc-tagged mFZD5 and FLAG-tagged RNF43 (WT). (**b**) FLAG-tagged RNF43 and its mutants were co-expressed with Myc-tagged mFZD5 in HEK293T cells. Subcellular localization of RNF43 and FZD5 was observed by confocal microscopy. (**c**) Fluorescence intensity of internalized FZD5 was quantified using Image J. Error bars represent ± SD. *P* value was determined by Student’s *t* test.

To further visualize the effects of RNF43 mutations on the subcellular distribution and expression of mFZD5, we co-transfected C-terminal FLAG-tagged WT RNF43 or RNF43 truncating mutations with mFZD5-Myc in HEK293T cells. As shown in Fig. 4a, mFZD5-Myc was expressed on the cell surface, in accordance with a previous study^5^. After co-transfection with FLAG-tagged WT RNF43, the expression of mFZD5-Myc on the cell surface was decreased and co-localized with WT RNF43 in the cytoplasm (Fig. 4b). However, in cells co-transfected with R117fs, mFZD5-Myc was localized on the cell surface. This was not observed when other FLAG-tagged RNF43 mutants were co-transfected with mFZD5-Myc (Fig. 4b, c). We also observed subcellular localization of WT RNF43 and its mutants. WT RNF43 has been detected at the cell surface by mass spectrometry and flow cytometry^5,12^. In this study, we observed subcellular localization of WT RNF43 and its mutants by fluorescence microscopy. RNF43 was localized at the cell surface and cytoplasm, although it was not as distinct as FZD5 localized at the cell surface. R337* and Q409fs mutants were clearly localized at the cell surface and in the cytoplasm upon co-expression with FZD5. Therefore, we overexpressed WT RNF43 and its mutants not tagged with flag (Supplementary Fig. S3). WT RNF43 was mainly localized in the cytoplasm, but also at the cell surface. R337* and Q409fs were localized at the cell surface.

### Extracellular interaction of RNF43 truncated mutants and FZD

**Figure 5:**
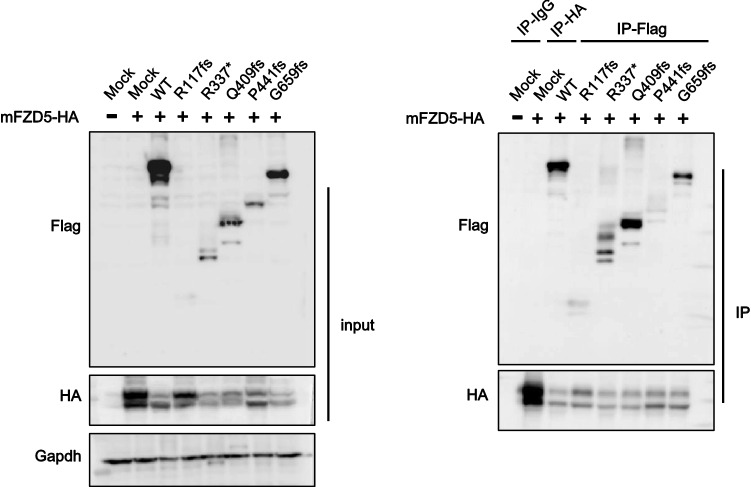
Co-immunoprecipitation of RNF43 and its mutants with FZD5. HEK293 cells were transfected with the indicated constructs and immunoprecipitated with control IgG or FLAG antibody. Immunoprecipitation and total cell lysates were immunoblotted with the indicated antibodies.

The protease-associated (PA) domain of RNF43 interacts with FZD5, resulting in the suppression of Wnt/β-catenin signaling^6^. Therefore, we co-transfected C-terminal FLAG-tagged RNF43 mutants or WT RNF43 with mFZD5-HA in HEK293T cells, followed by co-immunoprecipitation to determine if R117fs, R337*, Q409fs, P441fs, and G659fs interact with FZD5. We confirmed the interaction between FZD5 and WT RNF43 by co-immunoprecipitation, and predicted that the RNF43 truncated mutants, except R117fs, would interact with mFZD5 (because R117fs contains the partial region of the PA domain) (Fig. 1a). However, the co-immunoprecipitation results showed that all of the mutants, including R117fs, interacted with mFZD5 (Fig. 5).

### RNF43 R117fs mutant does not mediate FZD5 ubiquitination

**Figure 6:**
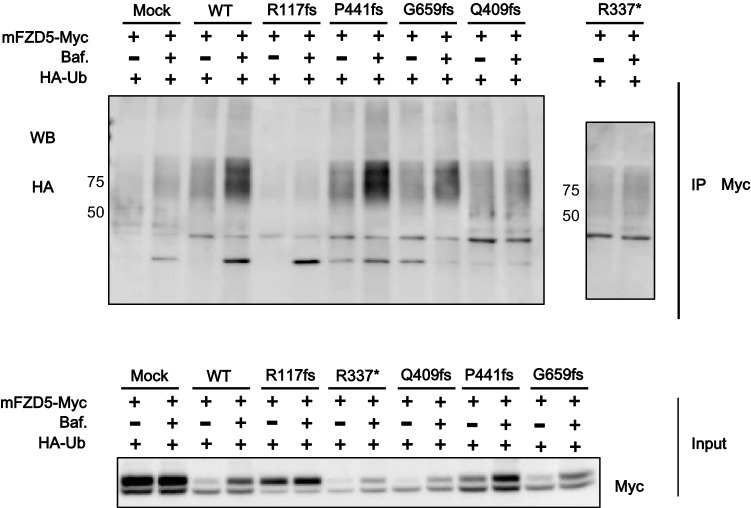
The RING domain of RNF43 is required for RNF43-mediated FZD5 ubiquitination. HEK293T cells were transfected with Myc-FZD5, FLAG-RNF43, or HA-ubiquitin as indicated. R117fs mutant, which does not contain the RING domain, suppressed the ubiquitination of FZD5. WT and mutant RNF43 increased the ubiquitination of FZD5.

Next, we investigated whether the RNF43 truncated mutants can mediate the ubiquitination of FZD5. As shown in Fig. 6, overexpression of WT RNF43 promoted the ubiquitination of mFZD5-myc, and degradation was inhibited by lysosomal inhibition with bafilomycin A1. Similar effects were observed upon co-transfection of mFZD5 with the RNF43 mutants, with the exception of R117fs, which does not contain the RING domain or suppress FZD5 ubiquitination. Taken together, these data showed that the R117fs mutant lost its negative regulator function because it lacks the RING finger domain, although it was able to interact with FZD5. Therefore, R117fs was unable to ubiquitinate mFZD5, resulting in surface expression of mFZD5 (Fig. 4b).

## Discussion

The components of the Wnt signaling pathway, such as APC and β-catenin, are frequently mutated in tumors^13,14^, leading to constitutive activation of β-catenin and abnormal activation of Wnt signaling. Therefore, the development of Wnt/β-catenin pathway inhibitors is very important for cancer therapies. The recent cancer genome atlas project and whole-genome sequencing analysis showed that genes associated with Wnt signaling are frequently mutated in gastric tumors^9,15^. Regulation of FZD receptor expression also influences Wnt signaling and plays an important role in cancer^13^. Several FZD receptors are upregulated in human tumors and cell lines^16–18^; therefore, turnover of Wnt receptors and cytoplasmic components of Wnt/β-catenin signaling is a critical regulatory mechanism in the Wnt signaling pathway. Ubiquitin-mediated degradation of Wnt receptors^19^, dishevelled^20^, Axin^21^, APC^22^, and β-catenin^23^ plays an important role in the regulation of Wnt signaling^4,6^. Ubiquitin-mediated protein degradation occurs in a three-step process via the sequential action of ubiquitin-activating (E1), ubiquitin-conjugating (E2), and ubiquitin-ligating (E3) enzymes^24^. RNF43, a cell surface transmembrane E3 ubiquitin ligase, shuts off the Wnt signaling pathway by promoting the ubiquitination and lysosomal degradation of FZD^5^. Thus, loss of function of RNF43 results in abnormal Wnt signaling. In this study, we demonstrated that the R117fs mutation led to loss of negative regulation of Wnt/β-catenin signaling, whereas other truncating mutations, including Q409fs and G659fs, maintained the function of RNF43.

WT RNF43 overexpression leads to the internalization of FZD5 localized at the cell surface, followed by termination of Wnt/β-catenin signaling^5,25^. In this study, we confirmed that FZD5 is localized at the cell surface in the absence of RNF43. However, FZD5 expression on the cell surface was decreased and localized in the cytoplasm in the presence of WT RNF43. This was not observed when the R117fs mutant lacking the RING domain was co-expressed with FZD5, while FZD5 expression at the cell surface was decreased upon co-expression with other mutants (Fig. 4). Therefore, the R117fs mutant promoted the accumulation of FZD5 at the cell surface, resulting in activation of the Wnt/β-catenin signaling pathway (Fig. 1c). This was also seen with ZNRF3, a cell surface transmembrane E3 ubiquitin ligase that is a functional homolog of RNF43. The ZNRF3 mutant lacking the RING domain upregulates cell surface expression of FZD^26^. These results suggest that the RING domain of RNF43 is important for regulation of Wnt/β-catenin signaling. We also observed subcellular localization of WT RNF43 and its mutants by fluorescence microscopy. WT RNF43 was mainly localized in the cytoplasm, but also at the cell surface. R337* and Q409fs were localized at the cell surface. Previous fluorescence microscopy studies have shown that WT RNF43 is localized in the endoplasmic reticulum, membrane, and nuclear envelope^6,27^. More studies are needed to understand the abnormal subcellular localization of mutants observed by fluorescence microscopy.

In our study, R117fs was a positive regulator of Wnt/β-catenin signaling; the other truncated mutants were negative regulators. We detected protein expression of FZD5 by western blotting. Overexpression of WT RNF43 decreased the mature form of FZD5; however, R117fs had no effect on the protein expression of FZD5, in accordance with the luciferase reporter assay results. The PA domain is present in various transmembrane proteins and mediates protein–protein interactions^28,29^. Interaction of the extracellular PA domain of RNF43 with FZD induced ubiquitination of FZD by its RING domain^6^. The R117fs mutant has a partial PA domain (Fig. 1a). In this study, co-immunoprecipitation showed that R117fs weakly interacted with FZD5 (Fig. 5). However, fluorescence microscopy and western blotting showed that overexpression of R117fs had no effect on FZD5 expression on the plasma membrane or the protein level (Figs. 3, 4). Also, ubiquitination of FZD5 was not mediated by R117fs (Fig. 6). These results suggest that R117fs, which has no RING domain, cannot downregulate surface expression of FZD5 by ubiquitin-mediated regulation, and that the RING domain is essential for regulation of FZD5 expression.

Wnt undergoes posttranslational acylation mediated by Porcupine^30^. LGK974 is a Porcupine inhibitor that specifically inhibits Wnt secretion and is currently under clinical evaluation^11^. Previous studies have suggested that Porcupine inhibitors, including LGK974 and IWP-2, significantly inhibit the proliferation of pancreatic cancer cell lines and the tumor growth of patient-derived xenografts with inactivating RNF43 mutations^11,12,31^. In this study, LGK974 effectively inhibited the Wnt/β-catenin signaling induced by R117fs and P441fs (Fig. 2). Therefore, it appears that R117fs and P441fs mutations lead to loss of the negative regulator function of RNF43, although P441fs acts as a partial positive regulator. R117fs, which has no RING domain, could not downregulate the surface expression of FZD5 by ubiquitin-mediated regulation. These two mutants were sensitive to the porcupine inhibitor LGK974 in vitro It is presumed that further verification is needed to determine whether LGK has therapeutic utility in cancers with R117fs and P441fs mutations. More studies are needed to identify cancer cell and mutant types that are sensitive to Porcupine inhibitors, as RNF43 mutations have been found in a variety of cancers such as pancreatic, stomach, ovary, and colon cancers^9,32–34^.

## Materials and methods

### Plasmids

Expression plasmids encoding full-length untagged and wild-type (WT) FLAG-tagged RNF43 were generated by PCR subcloning. Expression plasmids with point mutations were constructed by the introduction of these mutations by site-directed mutagenesis (Stratagene, La Jolla, CA, USA) according to the manufacturer’s instructions. Hemagglutinin (HA)-tagged ubiquitin and Renilla constructs were kindly provided by Dr. Kim (Ulsan National University of Science and Technology, Ulsan, Korea). The pLEX307-mFzd5 plasmid was a gift from Henry Ho (Addgene plasmid #102867; Addgene, Watertown, MA, USA)^35^. mFZD5-HA and mFZD5-Myc were amplified by PCR using pLEX307-mFZD5 as a template. All of the expression plasmids generated were reconfirmed with sequencing. M50 Super 8 × TOPflash (Addgene plasmid #12456) and M51 Super 8 × FOPflash (TOPflash mutant) (Addgene plasmid #12457) were a gift from Randall Moon^36^. pcDNA-Wnt3A was a gift from Marian Waterman (Addgene plasmid #35908)^37^.

### Cell culture and immunofluorescence

HEK293T cells were cultured in Dulbecco’s modified Eagle’s medium supplemented with 10% fetal bovine serum and 1% penicillin/streptomycin at 37 °C in 5% CO_2_. HEK293T cells were seeded on laminin-coated glass coverslips. At 24 h after seeding, cells were co-transfected with WT FLAG-tagged RNF43 and various FLAG-tagged mutants or mFZD5-Myc using polyethyleneimine (Polysciences, Inc., Warrington, PA, USA)^38^. At 48 h after seeding, cells were fixed in 4% paraformaldehyde, permeabilized with 0.2% Triton X-100 in phosphate-buffered saline (PBS) for 15 min at room temperature, and incubated with 1% bovine serum albumin to block nonspecific binding sites. Cells were labeled overnight at 4 °C with the following primary antibodies: anti-Myc (9E10; Thermo Fisher Scientific, Waltham, MA, USA), anti-FLAG (Millipore, Burlington, MA, USA), and anti-RNF43 (C252) (Dr. Kwon, Hallym University, Chuncheon, Korea). The cells were then incubated for 1 h at room temperature with Alexa Fluor-conjugated secondary antibodies, followed by staining with 4′,6-diamidino-2-phenylindole for 30 min. Finally, immunofluorescence was observed by confocal microscopy (Zeiss, Oberkochen, Germany).

### Luciferase reporter assay

Luciferase assays were performed using the Dual-Luciferase Reporter Assay System (Promega, Madison, WI, USA) according to the manufacturer’s protocol. Briefly, HEK293T cells were transiently co-transfected with 100 ng WT RNF43 or RNF43 truncating mutations with 1 ng Renilla, 100 ng Wnt3A, and 100 ng of either TOP or FOPflash luciferase reporter. After 48 h, cells were lysed with passive lysis buffer and pelleted in a microcentrifuge. The luciferase activity of each lysate was measured using the Lumat LB 9507 luminometer (Berthold Technologies, Oak Ridge, TN, USA) and normalized to the Renilla signal. All samples were measured in triplicate in each of three independent experiments. None of the constructs displayed significant FOPflash activity. LGK-974 was purchased from AdooQ Bioscience (Irvine, CA, USA).

### Immunoprecipitation and western blotting

HEK293T cells were transfected with the expression plasmids and treated overnight with bafilomycin A1 (10 nM; ApexBio, Houston, TX, USA) or MG132 (10 μM; Sigma, St. Louis, MO, USA). Then, transfected HEK293T cells were harvested and lysed in lysis buffer (50 mM Tris, pH 7.5, 150 mM NaCl, 0.5% Triton X-100, 5 mM EDTA, 1 mM DTT, and protease inhibitors). Whole-cell lysates were centrifuged at 4 °C, and supernatants were incubated with anti-FLAG (Sino Biological, Beijing, China), anti-Myc (Santa Cruz Biotechnology, Dallas, TX, USA), or anti-HA antibody (Santa Cruz) for 4 h, and with Protein G Agarose (Roche, Basel, Switzerland) for 12 h at 4 °C. After centrifugation, the Protein G Agarose beads were washed several times with PBS and the immunoprecipitated proteins were eluted from the beads with sample buffer. Protein samples were resolved by sodium dodecyl sulfate–polyacrylamide gel electrophoresis and electrotransferred to nitrocellulose membranes. Membranes were probed with anti-FLAG M2-HRP (1:1,000; Sigma), anti-HA-HRP (1:1,000; Roche), anti-ubiquitin (Santa Cruz), or anti-Myc (Thermo Fisher Scientific). Proteins were detected using Signal Light ECL Substrate (JUBIOTECH, Daejeon, Korea) and quantified with ImageJ software (NIH, Bethesda, MD, USA). Images were captured using the Amersham Imager 600 system (GE Healthcare Life Sciences, Marlborough, MA, USA). The protein levels were quantified using Image J (Java 1.8.0_172, imagej.nih.gov/ij/download.html).

## Supplementary Information

Supplementary Information 1.

Supplementary Information 2.
