# Supplementary material for: RNF43 R117fs mutant positively regulates Wnt/β-catenin signaling by failing to internalize FZD expressed on the cell surface
Source: Sci Rep. 2022 Apr 29;12:7013. doi: 10.1038/s41598-022-10868-8 (PMC9054848; doi:10.1038/s41598-022-10868-8)
Supplement: Supplementary file 1 — Supplementary Information 1. [file 41598_2022_10868_MOESM1_ESM.docx]

**Supplementary Figure Legends**

**Supplementary Figure 1. Distribution and type of RNF43 mutants in GC.** (a) Distribution and type of RNF43 mutations in GC identified in The Cancer Genome Atlas. (b) Distribution and type of RNF43 mutants in GC identified in our study.

**Supplementary Figure 2.** Expression of C-terminal FLAG-tagged WT-RNF43 and truncating mutations. The ubiquitination of RNF43 constructs was evaluated with MG132 treatment.

**Supplementary Figure 3.** Confocal immunofluorescence microscopy of WT RNF43 and RNF43 mutant constructs. R117fs mutant had no signal because the anti-RNF43 antibody recognizes amino acids 252–276 of human RNF43. White arrows indicate WT RNF43 on plasma membrane.

**Figure S1**

**Figure S2**

**Figure S 3**
