# Supplementary material for: RNF43 R117fs mutant positively regulates Wnt/β-catenin signaling by failing to internalize FZD expressed on the cell surface
Source: Sci Rep. 2022 Apr 29;12:7013. doi: 10.1038/s41598-022-10868-8 (PMC9054848; doi:10.1038/s41598-022-10868-8)
Supplement: Supplementary file 2 — Supplementary Information 2. [file 41598_2022_10868_MOESM2_ESM.docx]

**Supplementary Figure**. Full length blots of immunoblot analysis.

Two membranes were captured together using the Amersham Imager 600 system

Two membranes were captured separately using the Amersham Imager 600 system

Two membranes were captured together using the Amersham Imager 600 system

The two membranes were captured separately using the Amersham Imager 600 system


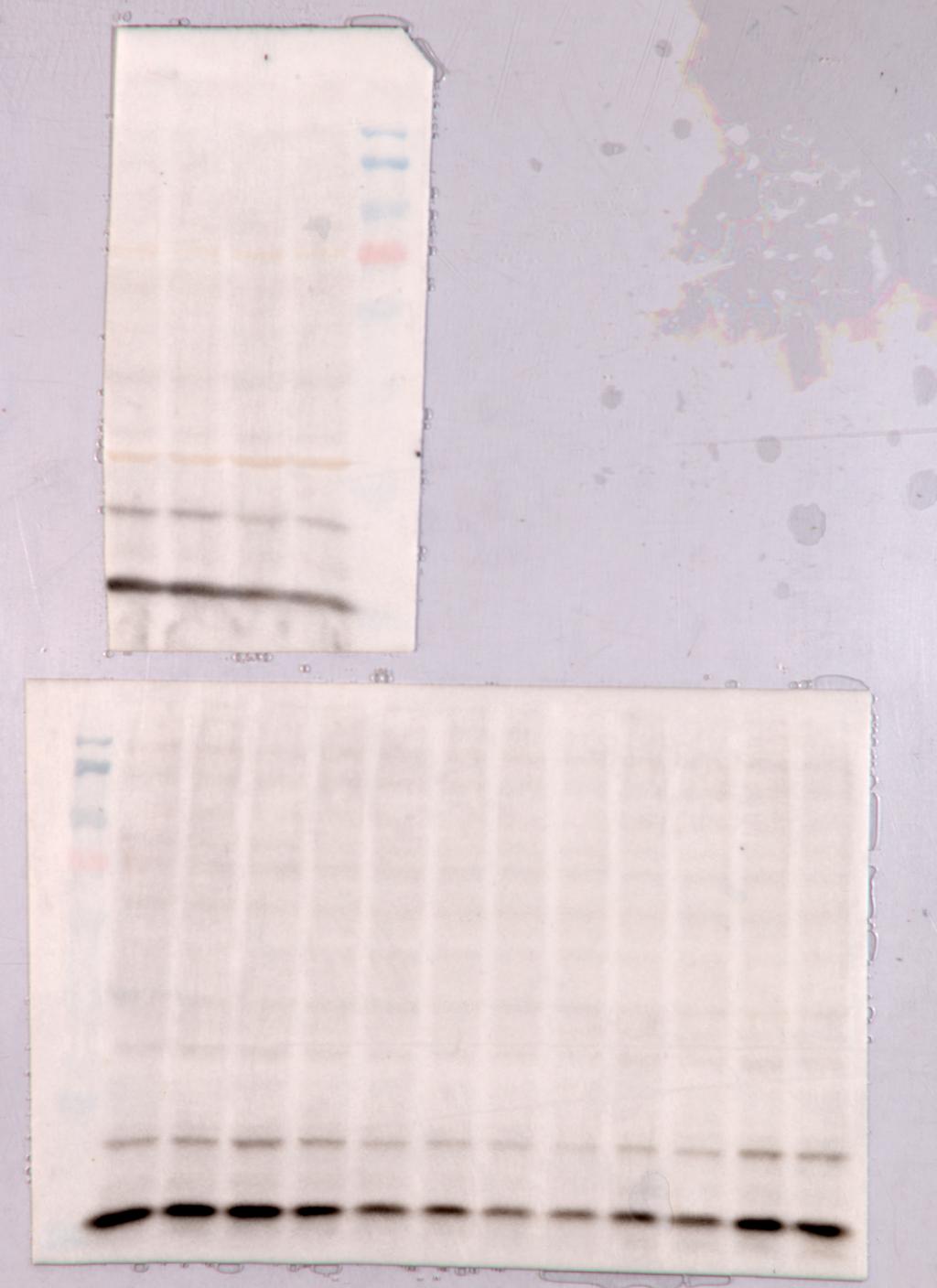

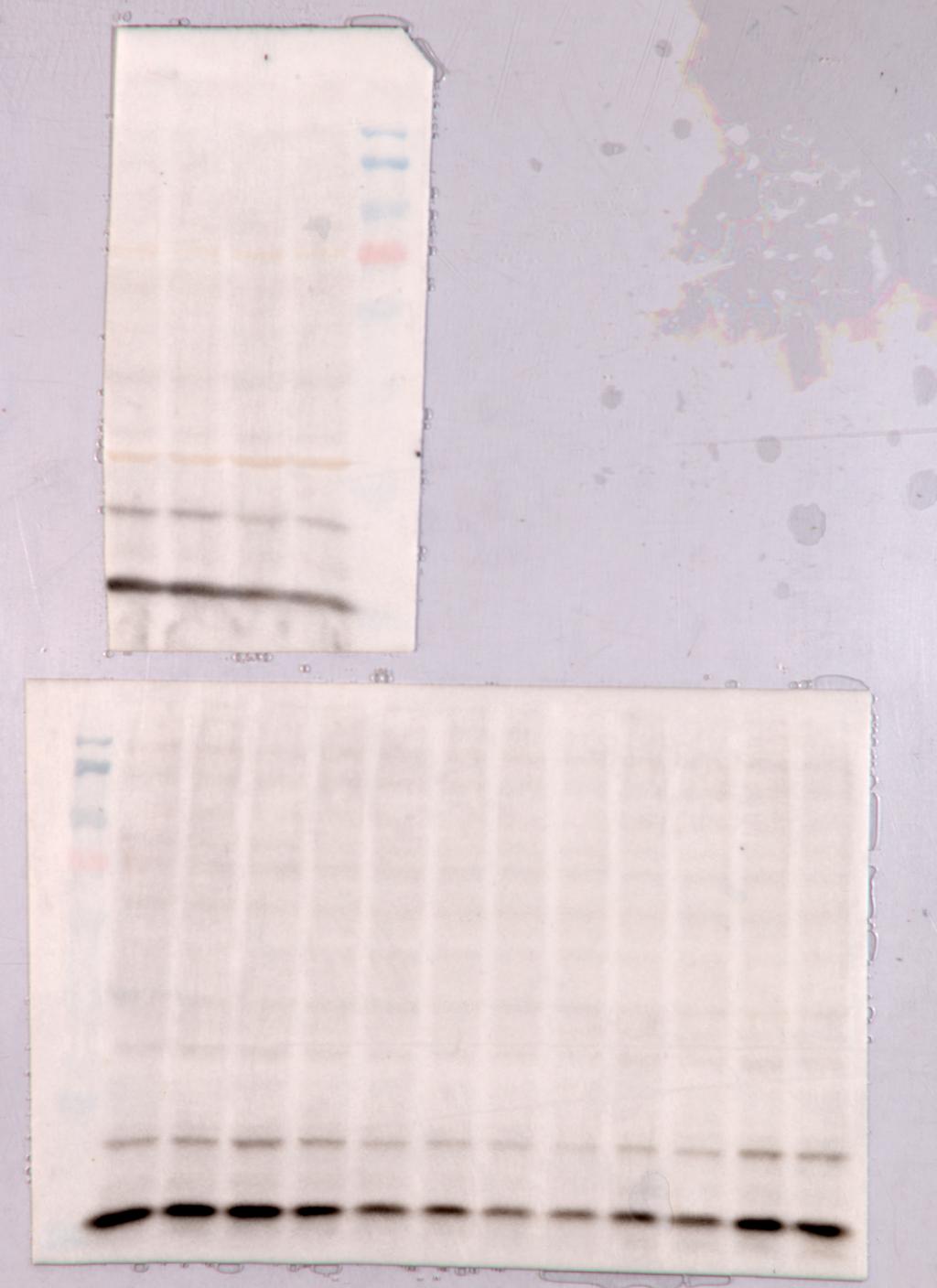


Two membranes were captured together using the Amersham Imager 600 system
